# Restored in vivo-like membrane lipidomics positively influence in vitro features of cultured mesenchymal stromal/stem cells derived from human placenta

**DOI:** 10.1186/s13287-017-0487-4

**Published:** 2017-02-07

**Authors:** Alexandros Chatgilialoglu, Martina Rossi, Francesco Alviano, Paola Poggi, Chiara Zannini, Cosetta Marchionni, Francesca Ricci, Pier Luigi Tazzari, Valentina Taglioli, Philip C. Calder, Laura Bonsi

**Affiliations:** 1 Remembrane Srl, Imola, Italy; 2 Department of Experimental, Diagnostic and Specialty Medicine, Unit of Histology, Embryology and Applied Biology, University of Bologna, Via Belmeloro 8, 40126 Bologna, Italy; 3 Department of Morphology, Surgery and Experimental Medicine, Section of Pathology, Oncology and Experimental Biology, University of Ferrara, Ferrara, Italy; 4 Department of Experimental, Diagnostic and Specialty Medicine, Unit of Nephrology, Dialysis and Renal Transplant, St, Orsola-Malpighi University Hospital, Via Massarenti 9, 40138 Bologna, Italy; 5 Service of Immunohematology and Transfusion Medicine, St. Orsola-Malpighi University Hospital, Via Massarenti 9, 40138 Bologna, Italy; 6 Laboratory of Molecular Biology, Institute of Cardiology, Department of Experimental, Diagnostic and Specialty Medicine, St. Orsola - Malpighi University Hospital, University of Bologna, Via Massarenti 9, 40138 Bologna, Italy; 7 National Institute of Biostructures and Biosystems at Ettore Sansavini Health Science Foundation ONLUS - Lab SWITH, Corso Garibaldi 11, 48022 Lugo (RA), Italy; 8 Human Development and Health Academic Unit, Faculty of Medicine, University of Southampton, Southampton, UK; 9 NIHR Southampton Biomedical Research Centre, University Hospital Southampton NHS Foundation Trust and University of Southampton, Southampton, UK

**Keywords:** Mesenchymal stromal cells, Stem cells, Membrane lipidomics, Membrane fatty acids

## Abstract

**Background:**

The study of lipid metabolism in stem cell physiology has recently raised great interest. The role of lipids goes beyond the mere structural involvement in assembling extra- and intra-cellular compartments. Nevertheless, we are still far from understanding the impact of membrane lipidomics in stemness maintenance and differentiation patterns. In the last years, it has been reported how in vitro cell culturing can modify membrane lipidomics. The aim of the present work was to study the membrane fatty acid profile of mesenchymal stromal cells (MSCs) derived from human fetal membranes (hFM-MSCs) and to correlate this to specific biological properties by using chemically defined tailored lipid supplements (Refeed®).

**Methods:**

Freshly isolated hFM-MSCs were characterized for their membrane fatty acid composition. hFM-MSCs were cultivated in vitro following a classical protocol and their membrane fatty acid profile at different passages was compared to the profile in vivo. A tailored Refeed® lipid supplement was developed with the aim of reducing the differences created by the in vitro cultivation and was tested on cultured hFM-MSCs. Cell morphology, viability, proliferation, angiogenic differentiation, and immunomodulatory properties after in vitro exposure to the tailored Refeed® lipid supplement were investigated.

**Results:**

A significant modification of hFM-MSC membrane fatty acid composition occurred during in vitro culture. Using a tailored lipid supplement, the fatty acid composition of cultured cells remained more similar to their in vivo counterparts, being characterized by a higher polyunsaturated and omega-6 fatty acid content. These changes in membrane composition had no effect on cell morphology and viability, but were linked with increased cell proliferation rate, angiogenic differentiation, and immunomodulatory properties. In particular, Refeed®-supplemented hFM-MSCs showed greater ability to express fully functional cell membrane molecules.

**Conclusions:**

Culturing hFM-MSCs alters their fatty acid composition. A tailored lipid supplement is able to improve in vitro hFM-MSC functional properties by recreating a membrane environment more similar to the physiological counterpart. This approach should be considered in cell therapy applications in order to maintain a higher cell quality during in vitro passaging and to influence the outcome of cell-based therapeutic approaches when cells are administered to patients.

## Background

In regenerative medicine, high therapeutic promises have been based on the possibility of stimulating ex vivo and in vitro expansion of stem cells and their differentiation into functional progeny that could regenerate injured tissues/organs in humans [1, 2]. Alterations in cell properties may occur during in vitro manipulation due to biochemical or biophysical changes from in vivo physiological conditions to in vitro ones [3, 4]. Without proper culture systems and protocols stem cells cannot grow normally outside the body and will gradually lose their multipotency or pluripotency (i.e., they will differentiate), and can undergo early senescence [5, 6]. Substrate surface chemistry and culture medium composition are the two main factors that researchers have spent a long time trying to optimize for in vitro cell culture [7]. The development of a fully defined and xeno-free system (i.e., chemically and physically defined) is required in stem/somatic cell culture to provide a “non-contaminated” cell population for cell therapy and tissue regeneration to eliminate safety concerns related to clinical use.

In this context, lipid metabolism is pivotal in stem cell physiology and it plays a central role in stem cell maintenance and differentiation [8–10]. However, the full understanding of stem cell lipid metabolism is still far away, but once achieved it could bring great advances in their handling and use. Kang and co-workers recently reviewed the preliminary efforts produced by the stem cell community in investigating the in vitro regulation of stem cell proliferation and differentiation by essential fatty acids and their metabolites [11].

Among stem cell populations, mesenchymal stromal cells (MSCs) draw the attention of the scientific community for their widespread properties, functions, and clinical applications [12, 13]. One of the most promising functions revolves around their interaction with the immune system, modifying its response [14, 15]. MSCs can be isolated from several fetal, perinatal, and adult tissues [16]. Placenta-derived tissues (i.e., placenta, fetal membrane, and umbilical cord) are interesting sources of MSCs for research purposes and clinical applications due to their readily availability and easy recovery without any ethical concerns [17]. These perinatal cells are able to proliferate in vitro for several passages [18] and present the classical MSC immunophenotype (CD44^+^, CD90^+^, CD73^+^, CD14^–^, CD31^–^, CD34^–^, CD45^–^) [19]. They can differentiate through the three classical mesodermal lineages (osteogenic, chondrogenic, and adipogenic) [20–22] and also into endothelial [23], hepatocytic [24], and myogenic [25] lineages, but present differences depending on the origin of the cells. Moreover, placenta-derived stromal cells have been demonstrated to preserve their plasticity alongside the maintenance of fetal tolerance due to their immunomodulatory properties [26–28].

These two biological characteristics could be useful for promising cell therapy approaches in regenerative medicine. Moreover, mesenchymal cells isolated from human placenta have been demonstrated to treat ischemia in an animal model [29] and reduce inflammation in pre-eclampsia-like features during pregnancy in an animal model [30]. The in vivo characteristics and properties of MSCs should be preserved in vitro in order to study and more effectively utilize MSCs.

Similar to the other typologies of stem cells, MSCs show a clear preference for ATP production through glycolysis, rather than by using glucose and fatty acid oxidation [31]. To date, only a few studies on MSC membrane lipids have been performed [32, 33], and membrane lipid composition during in vitro expansion of MSCs has not been studied systematically. Changes in the membrane fatty acid profile during MSC differentiation [34–36] and results obtained by lipid supplementation [37] show how membrane lipids play a crucial role in MSC metabolism, even if this is still mainly unexplored.

As shown elsewhere [38], an adequate integrative lipid supplementation is fundamental to improving the quality of in vitro cells and to modulate specific cellular properties of interest. In fact, in the majority of cases, cultured cells develop a membrane network that is altered and non-functional to their physiology, thus reducing their full potential.

In this work, we investigated how the membrane fatty acid composition of MSCs derived from human fetal membranes (hFM-MSCs) is modified by the in vitro culturing process. We showed that a tailored lipid supplement is able to maintain in vitro a membrane lipid pattern more similar to the physiological one. Finally, we explored how membrane lipid changes are able to impact on many relevant biological properties of in vitro hFM-MSCs and discuss potential implications of lipid monitoring and supplementation in cell therapy applications and related therapeutic approaches.

## Methods

### Isolation and cultivation of fetal membrane mesenchymal stromal cells

Term placentas were obtained by cesarean section from healthy donor mothers, after written informed consent and according to the policy of the local ethical committee. The study was approved by the local ethical committee (St. Orsola-Malpighi University Hospital Ethical Committee, protocol number 1645/2014, ref. 35/2014/U/Tess).

Fetal membrane samples were separated from the chorionic plate and washed in phosphate-buffered saline (PBS; Lonza, Walkersville, MD, USA) containing 1% penicillin-streptomycin solution (Lonza, Walkersville, MD, USA). For hFM-MSC isolation, small fragments of membranes were minced and then digested using a solution of 1 mg/ml collagenase type IV (Sigma-Aldrich Co., St. Louis, MO, USA) and 0.25% trypsin-EDTA (Lonza, Walkersville, MD, USA). The fragments were resuspended for 15 min, and then incubated for 15 min at 37 °C twice. Fetal bovine serum (FBS; Lonza, Walkersville, MD, USA) was added and a final centrifugation at 400 g for 10 min was performed. The pellet was resuspended and cells were plated in culture flasks in DMEM with high glucose (Lonza, Walkersville, MD, USA) and supplemented with 10% FBS, 2 mM l-glutamine and 1% penicillin-streptomycin solution.

### Membrane isolation following culture

Cells (7 × 10^6^) were collected in a 15-ml tube, centrifuged at 500 g for 5 min, and resuspended in 10 ml PBS. The wash was repeated five times in order to remove traces of medium and serum used during the culture process. Cells were then resuspended into 500 μl PBS and collected in a 1-ml tube, to which 500 μl sterile H_2_O were added. Cells were then centrifuged for 30 min at 15,000 g in a refrigerated centrifuge at 4 °C. The collected membranes were resuspended in 1 ml PBS:H_2_O (1:1) and washed five times following the same procedure.

### Fatty acid composition analysis

Cell membrane lipids were extracted with CHCl_3_/MeOH (2:1 vol/vol) and then incubated with 0.5 M KOH in methanol for 10 min at room temperature, thus trans-esterifying fatty acids linked by ester bonds to methanol to form fatty acid methyl esters (FAMEs). FAMEs were extracted with n-hexane and separated by gas chromatography in an Agilent 7820A GC System (Agilent Technologies, Santa Clara, USA) fitted with a 60 m × 0.32 mm DB23 capillary column, film thickness 0.25 μm, and a flame ionization detector (FID). Helium was used as a carrier gas at 2.54 ml/min and the spilt injector was used with a split ratio of 10:1. Injector temperature was 250 °C and detector temperature was 260 °C. The column oven temperature was maintained at 50 °C for 2 min after sample injection and was programmed for the following temperature gradient: 10 °C/min from 50 °C to 180 °C, 3 °C/min from 180 °C to 200 °C and holding at 200 °C for 6 min. The separation was recorded with G6714AA SW EZChrom Elite Compact (Agilent Technologies). FAMEs were identified by comparison with standards purchased from NuCheckPrep Inc. (Elysian, USA). FAMEs are expressed in weight %, based upon the precentage contribution of the peak area of each FAME in the chromatogram. To take into account the different signal of the detector for different molecules, a correction factor was applied to the experimental data coming from the integration of the chromatograms. The total of the peaks analyzed for each chromatographic run was 100.

Fatty acids are calculated as follows:

$$ \sum\ \mathrm{Saturated}\ \mathrm{Fatty}\ \mathrm{Acids}\ \left(\mathrm{SFAs}\right) = 14:0 + 15:0 + 16:0 + 17:0 + 18:0 + 20:0 + 22:0 + 23:0 + 24:0; $$

$$ \sum\ \mathrm{Monounsaturated}\ \mathrm{Fatty}\ \mathrm{Acids}\ \left(\mathrm{MUFAs}\right) = 16:1\mathrm{n}-7 + 18:1\mathrm{n}-9 + 18:1\mathrm{n}-7 + 20:1\mathrm{n}-9 + 22:1\mathrm{n}-9 + 24:1\mathrm{n}-9; $$

$$ \sum\ \mathrm{Polyunsaturated}\ \mathrm{Fatty}\ \mathrm{Acids}\ \left(\mathrm{PUFAs}\right) = 18:2\mathrm{n}-6 + 18:3\mathrm{n}-6 + 18:3\mathrm{n}-3 + 20:3\mathrm{n}-9 + 20:3\mathrm{n}-6 + 20:4\mathrm{n}-6 + 20:3\mathrm{n}-3 + 20:5\mathrm{n}-3 + 22:2\mathrm{n}-6 + 22:4\mathrm{n}-6 + 22:5\mathrm{n}-6 + 22:5\mathrm{n}-3 + 22:6\mathrm{n}-3; $$

$$ \sum\ \mathrm{Omega}-3 = 18:3\mathrm{n}-3 + 20:3\mathrm{n}-3 + 20:5\mathrm{n}-3 + 22:5\mathrm{n}-3 + 22:6\mathrm{n}-3; $$

$$ \sum\ \mathrm{Omega}-6 = 18:2\mathrm{n}-6 + 18:3\mathrm{n}-6 + 20:3\mathrm{n}-6 + 20:4\mathrm{n}-6 + 22:2\mathrm{n}-6 + 22:4\mathrm{n}-6 + 22:5\mathrm{n}-6. $$

### Refeed® supplements

Refeed® supplements (Remembrane Srl, Imola, Italy) are a completely defined combination of non-animal-derived lipids and antioxidants (NuCheckPrep Inc., Elysian, USA; Sigma Aldrich Co., St. Louis, MO, USA; Applichem an ITW Inc., Chicago, USA) solubilized in 1 ml ethanol (Sigma-Aldrich Co., St. Louis, MO, USA). One milliliter of Refeed® was diluted in 500 ml complete cell growth medium, with the resulting ethanol concentration being <1% (vol/vol) in the final medium. Refeed® composition is shown in Table 1. Starting from the first passage (P1), treated cells were supplemented with the full dose of Refeed®. During passage 0 (P0), the full dose was divided into three equal doses and supplemented at different times in order to allow cells to adapt to the supplement.

**Table 1:** Composition of Refeed® used for in vitro supplementation of human fetal membrane mesenchymal stromal cells

	Refeed®
Lipids	2.14
Antioxidants	0.62

Data are shown as the amount (mg) per 500 ml complete medium

### Immunofluorescence

Cells were seeded (2.5 × 10^4^) onto glass coverslips and fixed for 10 min in 2% paraformaldehyde at room termperature. After three washes in PBS, cells were permeabilized for 10 min with PBS 1% Triton (Triton X-100; Sigma-Aldrich, Co., St. Louis, MO, USA), then incubated in blocking solution 1× PBS 1% bovine serum albumin (BSA; Sigma-Aldrich, Co., St. Louis, MO, USA) for 30 min. Primary antibody anti-Vinculin (1:100; Chemicon, Temecula, CA, USA) diluted in blocking solution was added and incubated for 1 h at room temperature. After three washes in 1× PBS, secondary antibody anti-mouse Cy3 (1:100; Sigma-Aldrich, Co., St. Louis, MO, USA) was added diluted in blocking solution and incubated for 45 min at room temperature. Cortical actin was stained using FITC-Phalloidin (1:250; Sigma-Aldrich, Co., St. Louis, MO, USA) and added directly during secondary antibody incubation. Coverslips were mounted after washes in PBS in ProLong Gold Antifade Mountant with Dapi (Thermo Fisher Scientific, Monza, MB, Italy).

### Cell proliferation

hFM-MSCs from passages 1 to 8 were analyzed for their population doubling, population doubling time, and cumulative population doubling. Cells were seeded at an initial concentration of 5000 cells/cm^2^ in T25 culture flasks. At every passage, cells were harvested with 0.25% trypsin-EDTA solution for 3 min at 37 °C and counted with a hemocytometer by erythrosine B exclusion.

### Flow cytometry characterization

hFM-MSCs at the third passage were analyzed by flow cytometry (Attune NxT Flow Cytometer, Thermo Fisher Scientific) to verify their immunophenotypic profile. The antibodies used were: for hematopoietic markers (anti-CD14-allophycocyanin (APC), anti-CD34-fluorescein isothiocyanate (FITC) (Becton Dickinson), and anti-CD45-peridinin chlorophyll protein complex (PerCP) (Becton Dickinson)); for an endothelial-perivascular marker (anti-CD31-phycoerythrin (PE) (Becton Dickinson)); for stromal markers (anti-CD44-FITC (Biolegend), anti-CD73-PE (Becton Dickinson) and anti-CD90-PE (Biolegend)); and for the major histocompatibility complex class II (anti-HLA-DR-PE (Becton Dickinson)).

### Angiogenic differentiation

In order to induce cells through the angiogenic lineage they were cultured with and without Refeed® in DMEM containing 2% FBS with the addition of 50 ng/ml vascular endothelial growth factor (VEGF; Sigma-Aldrich Co., St. Louis, MO, USA) for 6 days, changing the medium every 2 days. As a negative control of angiogenesis, cells with and without Refeed® were cultured in standard medium (DMEM with 10% FBS). Then cells were fixed for flow cytometric analysis and the expression of FLT1 (VEGFR1; R&D system), KDR (VEGFR2), and von Willebrand factor (vWF; Abcam) was measured.

### Immunomodulation

In order to investigate the immunomodulatory properties of hFM-MSCs on activated peripheral blood mononuclear cells (PBMCs), they were plated in six-well plates at a density of 10,000 cells/cm^2^ and allowed to stabilize in culture for 1 day. PBMCs were isolated from the blood of healthy donors by density gradient centrifugation (Ficoll-Paque, Sigma-Aldrich) and co-cultured on hFM-MSC monolayers at a ratio of 10:1 in RPMI with 10% FBS (Lonza, Walkersville, MD, USA). PBMCs were activated by addition of phytohemagglutinin (PHA; 1 μg/ml; Sigma-Aldrich Co., St. Louis, MO, USA) and incubated for 72 h at 37 °C and 5% CO_2_. The negative control was PBMCs without PHA stimulation and the positive control consisted of PBMCs stimulated by PHA in the absence of hFM-MSCs. The immunomodulatory ability of hFM-MSCs was quantified by BrdU incorporation by activated PBMCs. After 72 h of co-culture between hFM-MSCs and PBMCs, the latter were recovered and seeded in a 96-well plate and then BrdU incorporation levels were quantified using a colorimetric immunoassay, according to the manufacturer’s instructions (Cell Proliferation ELISA, BrdU colorimetric kit, Roche, Basel, Switzerland). hFM-MSCs cultured with and without activated PBMCs were fixed for flow cytometric analysis. Finally, HLA-G (Abcam ab7904) expression of hFM-MSCs was measured to confirm the immunomodulatory phenotype.

### Statistical analysis

All the experiments were performed at least three times. Data are presented as means ± standard deviation and were analyzed by one and two-way ANOVA or *t* test using Graph Pad Prism software. The significance threshold was *p* < 0.05.

## Results

### Cultured hFM-MSCs have significantly different membrane fatty acid composition compared to their fresh uncultured counterparts

**Fig. 1:**
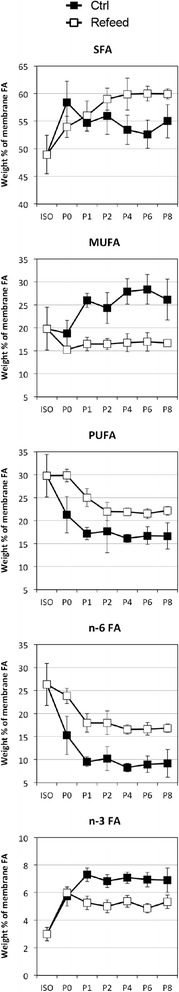
Partially preserved cultured hFM-MSC membrane fatty acid profile after Refeed® lipid supplementation. The main fatty acid parameters characterizing the membrane fatty acid profile of fresh uncultured hFM-MSCs (*ISO*), and how they are modified during normal (*Ctrl*) and Refeed®-supplemented in vitro culturing at different passages (*P0*, *P1*, *P2*, *P4*, *P6*, *P8*). Data are expressed in weight % of total membrane fatty acids and presented as means ± SD (*n* = 8 for ISO, *n* = 5 for in vitro cultivated hFM-MSCs). *FA* fatty acid, *MUFA* mono-unsaturated fatty acid, *n-3 FA* omega-3 fatty acid, *n-6 FA* omega-6 fatty acid, *PUFA* polyunsaturated fatty acid, *SFA* saturated fatty acid

Fresh uncultured hFM-MSCs showed variability in their membrane fatty acid composition, likely due to the genetic and lifestyle diversity of the donors. They had a membrane fatty acid composition mainly characterized by a high omega-6 fatty acid content, which represented more than 25% of total membrane fatty acids. The effect of culturing human hFM-MSCs in the traditional medium (DMEM + 10% FBS) on their membrane fatty acid composition was investigated and results are shown in Fig. 1. Data clearly demonstrate that significant differences arise during the first in vitro passages (mainly P0 and P1) between the membrane fatty acid profiles of in vitro cultivated hFM-MSCs and those of the corresponding fresh uncultured hFM-MSCs, considering both fatty acid sums (Fig. 1) and individual fatty acids (data not shown). Overall, cultured cells had lower proportions of polyunsaturated fatty acids (PUFAs) than freshly isolated cells. In particular, they showed a large drop in omega-6 fatty acids, counterbalanced by a marked increase in MUFA and omega-3 fatty acids (Fig. 1).

### Refeed® supplementation partially realigns hFM-MSC membrane fatty acid composition to that of their fresh uncultured counterparts

hFM-MSCs were cultured in the traditional medium (DMEM + 10% FBS) supplemented with specific Refeed® supplements, which are completely defined combinations of lipids and lipophilic antioxidants in ethanol (see Methods). Ethanol and antioxidants did not show any effect on cultured hFM-MSCs when tested as a negative control (data not shown). Culture with a tailored Refeed® formulation was able to partly prevent the changes induced by the traditional in vitro culture system and to restore the membrane fatty acid profile over time to one that better matched that of fresh uncultured hFM-MSCs (Fig. 1). In particular, Refeed® supplementation was able to partly reduce the loss of PUFA and omega-6 fatty acids in particular, while decreasing the accumulation of MUFA and omega-3 fatty acids. Individual fatty acids followed the same fluctuations (data not shown). Therefore, the membrane network of Refeed® supplemented hFM-MSCs better mimics that of fresh uncultured hFM-MSCs in its fatty acid composition and so most likely in its biophysical and functional properties.

### Isolation and proliferation

**Fig. 2:**
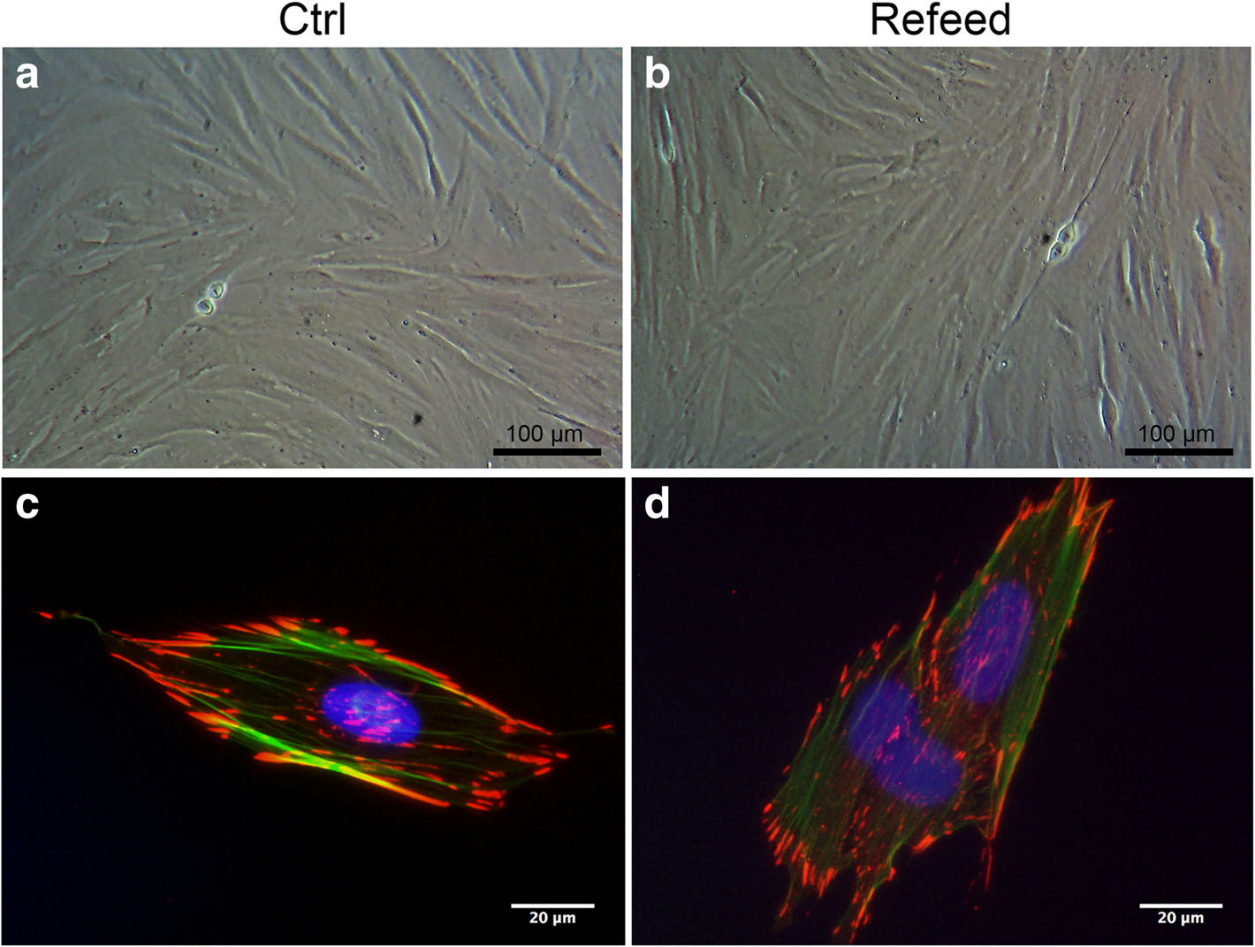
Unchanged hFM-MSC morphology after Refeed® lipid supplementation. Light microscopy images of expanded hFM-MSCs cultured in traditional medium (**a**; *Ctrl*) and with Refeed® supplementation (**b**). Immunofluorescence images for the cytoskeleton marker (phalloidin, *green*) and focal adhesion complexes (vinculin, *red*) of hFM-MSCs cultured in traditional medium (**c**) and with Refeed® supplementation (**d**). Nuclei were stained with Dapi (*blue*)

**Fig. 3:**
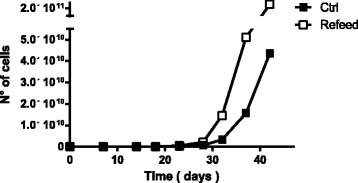
Increased hFM-MSC growth kinetics after Refeed® lipid supplementation. Theoretical in vitro expansion of hFM-MSCs. The number of cells was obtained considering the initial cell seeding (5000 cells/cm^2^) and the related cumulative population doubling. Control cells (*Ctrl*) are presented as *black squares* and cells supplemented with Refeed® as *white squares*

In order to evaluate the effect of Refeed® on cultured hFM-MSCs, cells were isolated and cultured in vitro with and without supplementation until passage eight (P8). Cells cultured with Refeed® showed a morphology similar to control cells, without lipid accumulation despite supplementation (Fig. 2a and b). In order to investigate also the cytoskeleton structure and the cell adhesion, in particular the focal adhesion complexes, an immunofluorescence for phalloidin and vinculin was performed. Cells cultured with Refeed® showed no changes to the cytoskeleton structure nor to the adhesion complex distribution compared to control cells (Fig. 2c and d). At each passage, cells were counted and population doubling, population doubling time, and cumulative population doubling were calculated. Figure 3 represents the theoretical number of cells obtained from initial cell seeding, valuated at cumulative population doubling obtained for each passage from 1 to 8. The increase in cell number, reflecting the rate of proliferation, was greater for cells cultured with Refeed® (Fig. 3).

### Immunophenotypic analysis

**Fig. 4:**
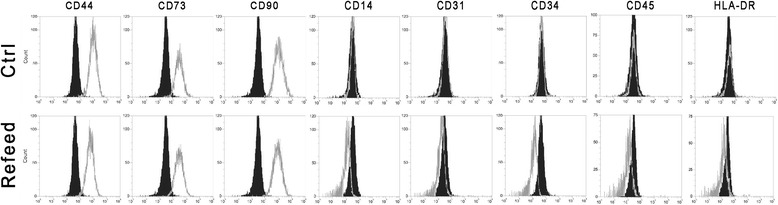
Unchanged hFM-MSC immunophenotype after Refeed® lipid supplementation. Representative flow cytometry analysis of mesenchymal (CD44, CD73, CD90), hematopoietic (CD14, CD34, CD45), and endothelial (CD31) markers, and major histocompatibility complex class II (HLA-DR). Unstained controls are presented as filled black histograms, the specific cell markers as grey histograms. *Ctrl* traditional medium

Cell surface antigens were assessed by flow cytometry analysis for a variety of markers associated with hematopoietic (CD14, CD34, and CD45), mesenchymal stromal (CD44, CD73, and CD90), and mature endothelial (CD31) cells. Finally, the major histocompatibility complex class II HLA-DR was also analyzed. Figure 4 shows that cells expanded for three passages in culture with Refeed® supplementation exhibited a similar immunophenotype to cells cultured in normal conditions, with high expression of stromal markers, and low expression of hematopoietic, endothelial markers, and HLA-DR.

### Angiogenic differentiation

**Fig. 5:**
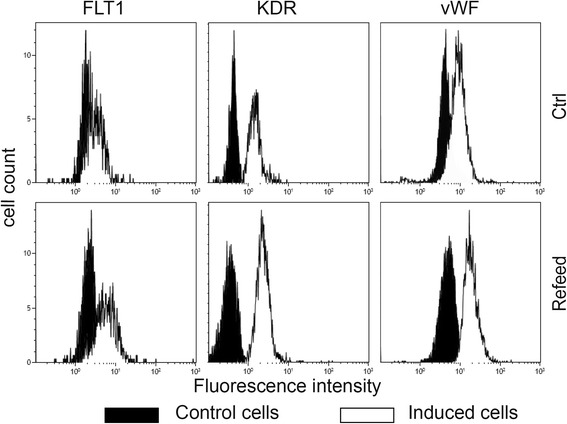
Improved hFM-MSC angiogenic differentiation after Refeed® lipid supplementation. Cells were induced with VEGF without (*Ctrl*) and with Refeed® supplementation. Representative flow cytometry analysis of the VEGF receptors FLT1 and KDR, and von Willebrand factor (*vWF*). Control cells are presented as *black histograms* and induced cells as *white histograms*

In order to understand the biological and functional effect of Refeed® we studied angiogenic differentiation in detail. Cells were induced for 6 days with VEGF and then fixed and analyzed by a flow cytometry procedure for the expression of FLT1, KDR, and vWF. As shown in Fig. 5, there was a clear increase of both VEGF receptors (FLT1 and KDR) and of the typical endothelial cell marker vWF expression in Refeed® supplemented cells after angiogenic stimulus.

### Immunomodulation

**Fig. 6:**
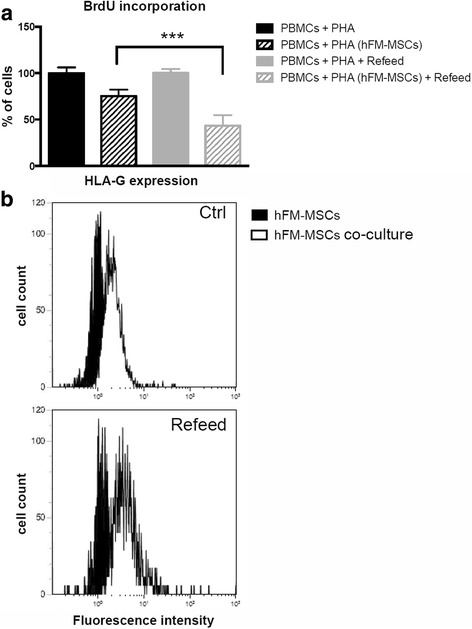
hFM-MSC enhanced immunosuppressive effect on activated peripheral blood mononuclear cells after Refeed® lipid supplementation. **a** Analysis of the BrdU incorporation into peripheral blood mononuclear cells (*PBMCs*) after co-culture with human fetal membrance mesenchymal stromal cells (*hFM-MSCs*) with and without Refeed® supplementation. Data are expressed as percentage of BrdU incorporation and presented as means ± SD (*n* = 3; ***P < 0.05). **b** Representative flow cytometry analysis of HLA-G expression on hFM-MSCs. Control cells are presented as *black histograms* and co-cultured cells as *white histograms. Ctrl* traditional medium, *PHA* phytohemagglutinin

Another important aspect of MSCs for clinical application is their ability to suppress the immune system. A co-culture of hFM-MSCs and PHA-activated PBMCs was performed and, after 3 days, BrdU incorporation and PBMC cell cycle were analyzed. Figure 6a shows how Refeed® supplementation increased the ability of hFM-MSCs to inhibit PBMC proliferation in vitro. In particular, activated PBMCs show a significantly lower BrdU incorporation when co-cultured with hFM-MSCs maintained in culture medium supplemented with Refeed® (43.27 ± 11.55%) compared to when they were cultured in standard conditions (75.25 ± 7.02%). These results suggest that hFM-MSCs have an immunomodulatory effect on stimulated PBMCs mediated by lymphoproliferative inhibition and that this effect is increased when cells are supplemented with Refeed®.

To support these data, we also analyzed the effect of Refeed® supplementation on the expression of HLA-G, one of the possible mediators of the effect previously described. In cells cultured in standard medium, HLA-G expression increased from 34.6 ± 4.1 to 55.9 ± 2.6% after co-culture with PHA-activated PBMCs whereas, in FM-MSCs supplemented with Refeed®, it increased from 46.2% ± 15.6 to 75.9 ± 10.7% after co-culture with PHA-activated PBMCs. Figure 6b shows that HLA-G expression on hFM-MSCs increases in co-culture with PBMCs and that the increase is greater in Refeed®-supplemented hFM-MSCs.

## Discussion

Membrane lipidomics is emerging as a core research field in stem cell biology [8–10]. Membrane lipids, including the fatty acid constituents, regulate many important aspects of stem cell physiology, stemness, and differentiation [11], although we are a long way from understanding the complete picture. In vitro culture conditions are considered important for generating safe and effective stem cells for clinical use in humans [5, 6]. However, current in vitro conditions for the culture of stem cells are still far from being optimized and much more needs to be done to achieve fully efficient in vitro cultivation processes in order for stem cells to be used in large-scale industrial processes of cell therapy and regenerative medicine applications [5, 6]. In this context, membrane lipidomics has not yet been considered as a key factor, and judicious lipid supplementation of in vitro stem cell cultures is limited and poorly thought out. This most likely reduces the potential of stem cell-based therapies.

Providing fatty acids to cultured cells is known to alter the fatty acid composition of their cell membrane and this is associated with changes in cell function [37, 38]. However, such effects are poorly studied and defined in stem cells. To investigate the impact that tailored lipid supplements, and thus different membrane lipidomics, could have on stem cells, we have chosen to study hFM-MSCs (perinatal cells derived from placental tissues) that are widely available, easy to recover, and raise no ethical issues [17]. These cells show stem cell-like features, such as high stemness and wide differentiation potential [20–25]. Moreover, these cells can be cultured in vitro for many passages and be expanded in sufficient number for clinical applications [18].

We showed that culture of hFM-MSCs resulted in marked changes in their fatty acid composition away from that seen in freshly isolated cells. A striking decrease in omega-6 fatty acids affected hFM-MSC membrane composition during the first in vitro passages (Fig. 1), probably due to the inability of hFM-MSCs to generate omega-6 fatty acids internally and to the shortage of omega-6 fatty acids provided by the serum. With the decrease in membrane fluidity caused by the drop in omega-6 fatty acid double bonds, cells increased MUFA synthesis through the upregulation of the Δ^9^-desaturase activity, thus lowering SFAs and raising MUFA content in the membrane system (Fig. 1). MUFAs partially counterbalanced the decrease in membrane fluidity, but not in membrane plasticity as they are not able to generate bioactive lipids as can omega-6 [39].

Furthermore, we identified that a tailored lipid supplementation allows hFM-MSCs to maintain a fatty acid composition that more closely resembles that of freshly isolated cells, so preventing membrane changes induced by in vitro cultivation (Fig. 1). This was associated with improved biological characteristics of the cultured hFM-MSCs. For example, the observed increase in hFM-MSC proliferation, without any morphology or immunophenotype alterations, indicates an overall improvement in cell welfare, probably due to a membrane network with a lipid composition more similar to that of the corresponding in vivo cells and to a better metabolism (Figs. 2, 3 and 4).

Increased angiogenic differentiation, documented by higher expression of a distinctive marker of endothelial cells (von Willebrand factor) (Fig. 5), is a sign of a greater cell plasticity, which suggests the likelihood of a greater efficiency in other differentiation processes. This is consistent with the higher presence of the two main receptors involved in angiogenesis (FLT-1 and KDR) in the plasma membrane of cells treated with Refeed® (Fig. 5). This suggests that the biological stimulus, in this case the differentiation stimulus, is more efficiently supported when the cells have a fatty acid composition more similar to the one they had in vivo. In addition to the higher number of these receptors in the membrane, it is likely that the signaling process also shows greater efficiency due to an increase in membrane domain stability provided by a different membrane order [40, 41].

The improvement of the immunomodulatory capacity of hFM-MSCs seen after culture in the presence of Refeed® (Fig. 6), albeit small, suggests an improvement also of the process of protein synthesis, protein folding, and trafficking, through which the receptors involved are transferred to the plasma membrane. In addition, a greater presence of bioactive lipids derived from omega-6 fatty acids, such as lipoxin A_4_, could be important in assuring a more optimal functioning of hFM-MSCs after culture in the presence of Refeed® [42].

Finally, experiments not reported here indicate a greater resistance of hFM-MSCs to the freeze/thaw processes (data not shown) and a marked improvement in the isolation efficiency of post-enzymatic digestion of hFM-MSCs treated with Refeed® (data not shown).

The effects described above originate from specific ad-hoc lipids that are used by the cell for the creation of a membrane network with different and more efficient features than seen if those lipids are not provided to the cells. A greater presence of PUFAs, particularly of omega-6 fatty acids, makes the membrane more fluid and more plastic [39], respectively due to the presence of a greater number of double bonds (compared with SFAs and MUFAs) and a greater presence of bioactive lipids. Greater efficiency in the process of trafficking, transmembrane protein folding, and signaling pathways could contribute to improve the global efficiency of the whole cell. Another interesting hypothesis comes from recent work [43] showing that an increase in membrane fluidity positively affects the solubility and diffusion of oxygen in membranes, ensuring a greater supply to mitochondria. This would improve oxidative metabolism.

From the results presented herein, culture of hFM-MSCs in the presence of the Refeed® lipid supplement creates in vitro conditions more similar to that seen in vivo, and contributes to a more efficient cell physiology and metabolism thus enabling the maintenance of many cellular responses and functions. It will be important to conduct a thorough study of the molecular changes that occur during the culture of these cells and how these changes are influenced by Refeed®. Among the most intriguing future perspectives, there is a possibility of investigating if and how the paracrine activity of these cells could possibly be influenced by an improved membrane lipid composition, and whether they could influence in turn the same or other cell types.

## Conclusions

It is now established that membrane lipidomics plays a fundamental role in cell physiology of stem cells and of MSCs in particular, although much still remains to be discovered. In order to achieve intensive use of these cells in cell therapy and regenerative medicine applications, it is necessary to make in vitro cultivation processes more efficient for them to be more suitable for their large-scale industrial use. It is in this context that membrane lipidomics and related tailored membrane lipid supplements can bring important advantages in the improvement of both in vitro amplification processes and the outcome of administering the same to patients, through an improvement in the quality of the cell and a realignment to in vivo conditions that enable the expression of the cells full potential. In this regard, culture of hFM-MSCs in the presence of the Refeed® lipid supplement creates in vitro conditions more similar to the in vivo one and contributes to a more efficient cell physiology and metabolism, thus enabling the maintenance of many cellular responses and functions.

## Abbreviations

FAME: Fatty acid methyl ester
FBS: Fetal bovine serum
hFM-MSC: Human fetal membrane mesenchymal stromal cell
MSC: Mesenchymal stromal cell
MUFA: Mono-unsaturated fatty acid
PBMC: Peripheral blood mononuclear cell
PBS: Phosphate-buffered saline
PHA: Phytohemagglutinin
PUFA: Polyunsaturated fatty acid
SFA: Saturated fatty acid
VEGF: Vascular endothelial growth factor
vWF: Von Willebrand factor
